# Self-Test Web-Based Pure-Tone Audiometry: Validity Evaluation and Measurement Error Analysis

**DOI:** 10.2196/jmir.2222

**Published:** 2013-04-12

**Authors:** Marcin Masalski, Tomasz Kręcicki

**Affiliations:** ^1^Department and Clinic of Otolaryngology, Head and Neck SurgeryWroclaw Medical UniversityWrocławPoland; ^2^Institute of Biomedical Engineering and InstrumentationWroclaw University of TechnologyWrocławPoland

**Keywords:** pure tone audiometry, computer-assisted instruction, self-examination

## Abstract

**Background:**

Potential methods of application of self-administered Web-based pure-tone audiometry conducted at home on a PC with a sound card and ordinary headphones depend on the value of measurement error in such tests.

**Objective:**

The aim of this research was to determine the measurement error of the hearing threshold determined in the way described above and to identify and analyze factors influencing its value.

**Methods:**

The evaluation of the hearing threshold was made in three series: (1) tests on a clinical audiometer, (2) self-tests done on a specially calibrated computer under the supervision of an audiologist, and (3) self-tests conducted at home. The research was carried out on the group of 51 participants selected from patients of an audiology outpatient clinic. From the group of 51 patients examined in the first two series, the third series was self-administered at home by 37 subjects (73%).

**Results:**

The average difference between the value of the hearing threshold determined in series 1 and in series 2 was -1.54dB with standard deviation of 7.88dB and a Pearson correlation coefficient of .90. Between the first and third series, these values were -1.35dB±10.66dB and .84, respectively. In series 3, the standard deviation was most influenced by the error connected with the procedure of hearing threshold identification (6.64dB), calibration error (6.19dB), and additionally at the frequency of 250Hz by frequency nonlinearity error (7.28dB).

**Conclusions:**

The obtained results confirm the possibility of applying Web-based pure-tone audiometry in screening tests. In the future, modifications of the method leading to the decrease in measurement error can broaden the scope of Web-based pure-tone audiometry application.

## Introduction

The development of Internet technologies combined with technological progress in the construction of personal computers, especially in terms of improving performance of sound cards, offer the possibility of conducting self-administered hearing tests at home. Moreover, in a group of elderly people who often suffer from hearing loss, there is an increasing number of Internet users. The validity of such hearing tests needs to be evaluated before applying the test results in the diagnostic and therapeutic process.

The remote examinations of hearing conducted with the use of PCs have been carried out for about 10 years [1]. These examinations can be divided into surveys [2-4] and examinations done with the use of sound signals. The sound signals can be generated through a dedicated external device connected to a PC, usually an audiometer [5,6] or a PC sound card [3,4,7-12]. Among hearing examinations, we can distinguish screening tests [3,7,11,12] and examinations such as pure-tone audiometry [4-6,8-10], which provide additional information.

The hearing screening tests done remotely with the use of a PC sound card are most often in the form of a speech-in-noise test [3,7,11,12]. The speech-in-noise test is preferred over surveys [3] as it improves the detection of hearing impairment among the population [7], especially after introducing a low-pass noise [12]. The evaluation of validity of pure-tone audiometry conducted in similar conditions, ie, with the use of a PC sound card and ordinary headphones [8-10] is ambiguous and depends on the adopted solutions, which include, eg, calibration, hearing threshold evaluation method, and presence or lack of a person supervising the test. In the supervised tests, the mean error concerning the determination of a hearing threshold on a specially constructed and calibrated PC-based device was below 2.3dB [8]. In unsupervised tests conducted after computer calibration performed by a person with good hearing, the greatest error occurred at the frequency of 2kHz and 4kHz and was −5.6dB and −5.1dB respectively, with standard deviation of 8.29dB and 6.9dB [10]. In unsupervised tests conducted without calibration, the maximum mean difference occurred at the frequency of 500Hz and was 11.3dB [9].

The potential application of pure-tone audiometry conducted on a PC depends on the value of the measurement error. The PC-based test will not substitute the clinical pure-tone audiometry. However, it can be applied for conducting self-administered check-ups in cases of limited access to clinical devices, eg, at the general practitioner. Alternatively, it can be used as an initial telemedical examination combined with a survey, which will help determine the direction of future treatment. The aim of the research was to identify the measurement error connected with determining the hearing threshold conducted by means of self-administered Web-based pure tone audiometry, as well as identify and analyze factors affecting its value.

## Methods

The hearing threshold evaluation was done in three series: (1) audiologist-performed tests on clinical audiometer at an audiology outpatient clinic, (2) self-administered Web-based tests on a specially calibrated computer at an audiology outpatient clinic under the supervision of an audiologist, and (3) self-administered Web-based tests conducted at home.

In series 1 and 2, 51 participants (32 men, 19 women), aged 11-60 years (the median age was 34) underwent examination. The research participants were selected from among the patients of an audiology outpatient clinic. The qualification criterion was the willingness to participate in the research, owning a PC, basic skills to operate it, and having an email account. 102 ears were examined from which 45 (44%) were ears without a hearing loss, ie, with the hearing threshold of 25dBHL and less, 17 (16.7%) were ears with hearing impairment below 40dBHL, 31 (30.4%) with hearing impairment above 40dBHL and below 70dBHL, and 9 (8.8%) were above 70dBHL. From the group of 51 patients examined in the first two series, series 3 was self-administered at home by 37 subjects (73%). The examinations in series 1 and 2 were conducted on the same day, and examinations from series 3 were conducted up to 196 days later (median of 9 days). In the case of failure to conduct test in series 3 in a time of 2-3 weeks, the patients were reminded by a phone call and then after about 1 month, they were reminded by an email.

### Tests in Clinical Settings

All tests from series 1 were made in an acoustic cabin with the use of clinical audiometer Interacoustic AD229e and headphones TDH-39. The calibration of the audiometer was conducted according to ISO 389-1:1998. The hearing threshold in pure-tone audiometry was determined with the use of the ascending method, according to ISO 8253-1:2010. The level of the tone was reduced in 10dB steps until no further response occurred, and then it was increased in 5dB steps until the subject responded. The threshold was defined as the lowest level at which responses occurred in at least half of the series of ascending trials with a minimum of two responses required at that level [13]. The examinations were conducted at the frequencies of 250Hz, 500Hz, 1kH, 2kHz, 4kHz, and 8kHz [13].

### Self-administered Web-Based Test

The examinations in series 2 and 3 were conducted on personal computers following their calibration. Both the calibration and the examination were performed at system volume set at the maximum level. The calibration consisted of determining the reference sound level by a person with good hearing. During calibration, two sounds differing in intensity were presented bilaterally in turns for 1 second at a frequency of 1kHz. The difference in sound intensity was stable and equalled 5dB. The task of the reference person was to set the volume in a way that ensured that only the louder of the two sounds presented was audible. The volume was controlled by means of a slider with the step of 1dB. The reference person could listen to the sound for unlimited time, adjusting the volume using the slider with 1dB step any number of times, in order to finally confirm the selected level with a button. The mean intensity of the two presented sounds determined the hearing level of the reference person and was marked 0dBRP-HL. The reference values at frequencies differing from the calibration frequency, ie, 1kHz, were calculated using the model based on the A-weighting filter [14].

The procedure of determining the hearing threshold during the test, both in series 2 and 3, differed from that applied during calibration. The task of the subject was to set the intensity of the presented pure tone modulated by sinusoidal envelope with a 2-second period at such a level that the sound was on the verge of audibility. Patients could listen to the sound for unlimited time and change the intensity of the presented sound signal by themselves using a slider with 5dB step any number of times, and then confirm the chosen level with a button. The hearing threshold was determined at frequencies as in series 1, ie, 250Hz, 500Hz, 1kHz, 2kHz, 4kHz, and 8kHz. For hearing thresholds below 0dBHL-RP, the value was assumed to be 0dBHL-RP. The time spent on calibrating and examinations was not recorded.

All the examinations in series 2 were conducted on a notebook Dell Vostro 1320 with operational system Microsoft Windows 7 and headphones Technics RP-F290 placed in an acoustic cabin of an audiology outpatient clinic and connected to the Internet. The calibration was repeated six times by 3 people whose hearing threshold in pure-tone audiometry at the calibration frequency was 0dBHL. Each person performed calibration twice. The final value of the calibration coefficient was determined as the mean of all values obtained from single calibrations and was the same for all examinations in series 2. Examinations were supervised by an audiologist, whose task was to train a patient and detect hisher mistakes, eg, changing the sides of headphones, omission of the frequency, or accidental marking of a threshold that was significantly different from the actual one.

Examinations in series 3 were conducted by the patients themselves on their own personal computers at home. Each examination was conducted on a different computer using different headphones. The test station was calibrated by other household members with no previous hearing problems. In that series, the hearing threshold of the reference person was not controlled. The trial participants were instructed to conduct the test at home in a quiet place, preferably in the evening or at night. Moreover, they were informed that the calibration should be performed by a person up to 35 years old, with no previous hearing problems.

## Results

### Comparison of the Results Between Series

**Figure 1:**
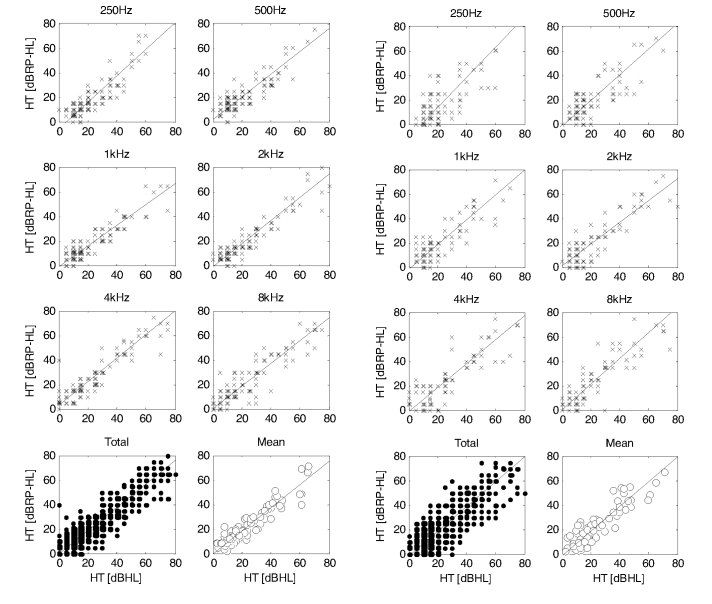
The relationship between hearing thresholds in series 1 and 2 (first two columns on left) and between series 1 and 3 (last two columns on right) calculated in a group of 51 and 37 subjects, respectively.

The hearing threshold determined in series 1 was compared to hearing thresholds from series 2 and 3. If there was no response at a given frequency, measurement was not taken into account for further calculations. Figure 1 presents relationships between the series: the relationship between hearing thresholds with division into frequencies, the relationship between total hearing thresholds, and that between the mean hearing thresholds calculated on the basis of the value obtained at all the examined frequencies.

The mean difference between thresholds in series, its standard deviation, Pearson’s correlation coefficient, and linear estimators (Table 1) were calculated for the relationships described above. Linear estimators were determined for Deming’s regression because the explanatory variable, which constitutes the hearing threshold in series 1, is also burdened with measurement error. The mean difference in the hearing threshold between series 1 and 2 and between series 1 and 3 was −1.54dB and −1.34dB respectively, with standard deviation 7.88dB and 10.66dB respectively, and Pearson’s correlation coefficients .90 and .84 respectively. In both comparisons, the lowest values of Pearson’s correlation coefficient were obtained for the frequency of 250Hz at the level of .88 and .69 respectively. The highest value of the standard deviation occurred at the frequency of 8kHz (8.88dB) and 500Hz (12.03dB) respectively. Pearson’s correlation coefficients calculated for the mean threshold were at the level of .94 and .89 respectively (Table 1).

The mean difference between thresholds in series, its standard deviation, Pearson’s correlation coefficient, and linear estimators of Deming’s regression were determined for the hearing loss below 40dBHL as well as greater than or equal to 40dBHL (Table 2). The division was made on the basis of the mean value of the hearing threshold in two comparable series. The mean difference in the hearing threshold did not exceed 2dB in any of the groups, and its standard deviation increases together with the increase in the hearing loss. Pearson’s correlation coefficients reflect changes in the standard deviation and reach low values due to narrow ranges of random variables compared to their standard deviations. For the same reason, the values of linear estimators deviate from the values established for the whole group.

On the basis of the obtained results, the sensitivity and specificity of noise-induced hearing loss detection was calculated, according to the criteria proposed in [15] adopted for the purposes of this paper. The noise-induced hearing loss was detected when the hearing threshold exceeded 30dB at one of the following frequencies: 500Hz, 1kHz, 2kHz, or 25dB at more than one, or when the hearing threshold exceeded 50dB at 4Hz. For series 2, the sensitivity was 0.92 with confidence interval of (0.81, 1.0) at *P*=.05, and the specificity was 0.96 with confidence intervals of (0.88, 1.0) at *P*=.05. For series 3, the respective values were sensitivity 0.89 (0.74, 1.0) and specificity 0.89 (0.76, 1.0).

### Analysis of the Measurement Error

This paper attempts to identify and assess the values of the sources of error in determining the hearing threshold of Web-based examinations. The literature data on standard deviation of the hearing threshold determined in test-retest examinations carried out in clinical settings are presented in Table 3. On the basis of these data, the value of the standard deviation of the hearing threshold determined in test-retest examinations was adopted for the further calculations at the level of 6dB.

**Table 1:** The mean difference *m* in the hearing threshold value between the series calculated on the basis of n data points collected on a group of N subjects, its standard deviation σ, Pearson’s correlation coefficient *r* and linear estimators *a*, *b* of Deming’s regression *y*=*ax*+*b* and corresponding confidence intervals CI at *P*=.05.

	f	n	*m*	σ	*r* (CI)	*a* (CI)	*b* (CI)
**Series 1 vs 2, N=51**							
	250Hz	100	−3.70	7.30	.88 (0.82, 0.92)	1.08 (1.06, 1.09)	−5.49 (−5.84, −5.13)
	500Hz	101	0.52	7.20	.88 (0.83, 0.92)	0.93 (0.92, 0.94)	2.09 (1.83, 2.34)
	1kHz	101	−4.59	6.91	.92 (0.88, 0.94)	0.85 (0.84, 0.86)	−1.16 (−1.39, −0.92)
	2kHz	101	−2.42	7.22	.93 (0.90, 0.95)	0.95 (0.94, 0.96)	−1.18 (−1.44, −0.91)
	4kHz	100	2.15	7.68	.93 (0.90, 0.95)	0.97 (0.96, 0.98)	2.88 (2.64, 3.12)
	8kHz	100	−1.20	8.88	.91 (0.86, 0.94)	0.93 (0.92, 0.93)	0.71 (0.47, 0.95)
	Total	603	−1.54	7.88	.90 (0.89, 0.92)	0.96 (0.96, 0.96)	−0.52 (−0.57, −0.48)
	Mean	99	−1.52	5.42	.94 (0.91, 0.96)	0.94 (0.93, 0.96)	−0.23 (−0.46, −0.01)
**Series 1 vs 3, N=37**							
	250Hz	71	−5.07	12.03	.69 (0.55, 0.80)	1.23 (1.19, 1.26)	−10.42 (−11.14, −9.70)
	500Hz	71	−0.35	10.43	.79 (0.68, 0.86)	1.05 (1.03, 1.08)	−1.57 (−2.08, −1.05)
	1kHz	72	−1.24	9.28	.85 (0.76, 0.90)	1.01 (0.99, 1.03)	−1.49 (−1.96, −1.02)
	2kHz	72	−1.58	10.46	.88 (0.81, 0.92)	0.90 (0.87, 0.92)	1.04 (0.48, 1.59)
	4kHz	72	−1.42	10.77	.87 (0.80, 0.92)	0.98 (0.96, 1.00)	−0.83 (−1.33, −0.33)
	8kHz	71	1.63	10.05	.88 (0.82, 0.93)	1.05 (1.04, 1.07)	0.31 (−0.11, 0.73)
	Total	429	−1.34	10.66	.84 (0.81, 0.86)	1.02 (1.01, 1.02)	−1.71 (−1.80, −1.62)
	Mean	68	−1.44	7.59	.89 (0.83, 0.93)	1.01 (1.00, 1.03)	−1.73 (−2.14, −1.33)

**Table 2:** The mean difference *m* in the hearing threshold *t* between the series calculated on the basis of n data points collected on a group of N subjects, its standard deviation σ, Pearson’s correlation coefficient *r* and linear estimators *a*, *b* of Deming’s regression *y*=*ax*+*b* and corresponding confidence intervals CI at *P*=.05 for the hearing threshold below 40dBHL as well as greater than or equal to 40dBHL.

Group		n	*m*	σ	*r* (CI)	*a* (CI)	*b* (CI)
**Series 1 vs 2, N=51**							
	*t*<40dBHL	498	−1.45	7.37	.74 (0.70, 0.78)	0.84 (0.84, 0.84)	1.24 (1.19, 1.30)
	*t*≥40dBHL	105	−1.99	9.88	.64 (0.51, 0.74)	1.07 (1.04, 1.09)	−5.67 (−7.04, −4.30)
**Series 1 vs 3, N=37**							
	*t*<40dBHL	339	−1.52	9.72	.58 (0.51, 0.65)	1.11 (1.10, 1.12)	−3.38 (−3.53, −3.22)
	*t*≥40dBHL	90	−0.64	13.67	.44 (0.25, 0.59)	0.66 (0.62, 0.69)	17.59 (15.56, 19.62)

**Table 3:** Standard deviations of the hearing threshold obtained in test-retest examination (literature data) (separate measurements were carried out for supra-aural headphones (series I, II, III) and in-ear headphones (IV, V, VI) and for the group of young (series I and IV), older (II, V) and the oldest (III, VI) subjects; N=number of subjects) [16-18].

Investigator	Series	N	250	500	1kHz	2kHz	4kHz	8kHz	Mean
Brown 1948 [16]		30	5.3	4.4	5.8	4.5	5.4	7.1	5.4
Erlandsson et al 1979 [17]^a^		10		6.3	6.2	4.7	5.3	10.4	6.6
Landry et al 1999 [18]	I	20	5.0	4.6	5.2	4.1	4.3	6.0	5.9
	II	10	8.5	7.9	5.2	6.4	9.5	6.9	
	III	10	5.9	5.4	6.4	2.1	6.7	7.1	
	IV	20	8.8	7.7	4.6	5.5	2.9	3.6	
	V	10	7.1	4.4	7.4	5.5	2.6	5.4	
	VI	10	9.3	4.6	3.3	5.3	9.0	9.4	
Mean			7.1	5.7	5.5	4.8	5.7	7.0	6.0

^a^Estimated by comparison with Békésy audiometry performed 5 times on a group of 10 subjects.

Knowing that the variance of the sum of the two independent random variables *X* and *Y* is the sum of their variance (see Multimedia Appendix 1), the standard deviation for a single measurement of the hearing threshold in clinical settings σ_*clin*_ was calculated at the level of 4.25dB by dividing the σ_*2clin*_ by the square root of two (see Multimedia Appendix 2).

Therefore, assuming the variability of the hearing threshold measurement in series 1 σ_(i)_ at the level of literature data σ_*clin*_, the standard deviation of the measurement error in Web-based hearing tests for series 2 σ_(ii)_ was calculated at 6.64dB based on the value σ_(i)_ and the value σ_(i)(ii)_, which is the standard deviation of the hearing threshold difference between the values determined in series 1 and 2. Similarly, for series 3, we obtain the standard deviation σ_(iii)_ equal to 9.78dB (see Multimedia Appendix 3).

In series 2, the standard deviation of the measurement error depends mainly on the standard deviation of the error connected with the procedure of determining threshold value σ_*proc*_. In series 3, apart from the error connected with the procedure of determining the threshold value, we can distinguish other sources of error influencing the standard deviation: the standard deviation of the calibration error σ_*cal*_, which is the reference sound level evaluation error at the calibration frequency, the frequency nonlinearity error σ_*nonlin*_, which is the difference between the actual reference sound level and that set by the model, the gain error σ_*gain*_, and the error connected with background noise σ_*noise*_ (see Multimedia Appendix 4).

Of course in series 2, the calibration error, frequency nonlinearity error, and gain error were the same in all tests and did not influence the value of standard deviation, while the error connected with background noise can be omitted since the tests were conducted in an acoustic cabin.

**Figure 2:**
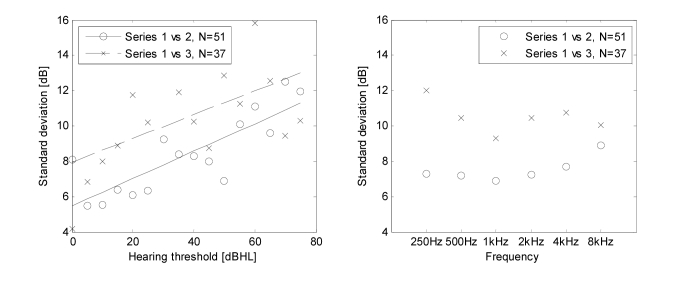
Standard deviation of the hearing threshold differences between the series calculated for the group of N subjects in relation to the hearing threshold evaluated in series 1 (left) and the frequency (right).

Figure 2 shows the standard deviation of threshold differences between series in relation to the hearing threshold for series 1 and the frequency. The lowest values of standard deviations are observed in small hearing losses, ie, for those measurements that should be most affected by the background noise. Therefore, we can assume that the error connected with background noise is significantly smaller than other components of the measurement error. In the following considerations, the standard deviation of error connected with background noise was omitted (σ_*noise*_≈0dB), both for series 2 and for series 3.

With the increase in intensity, increment of the standard deviation of measurement error in series 3 is slightly smaller than in series 2. In series 2, the standard deviation of the gain error equals zero since all tests are done on the same set. The lack of increase in standard deviation in series 3 above the values observed in series 2 indicates a negligible effect of the gain error on the measurement values (σ_*gain*_≈0dB).

The standard deviation of the calibration error σ_*cal*_ can be estimated by comparing the standard deviations for series 2 and 3 at the calibration frequency, ie, 1kHz. Since the calibration is performed on the same set as the test, the error connected with the nonlinearity of the frequency response at the calibration frequency is zero (_1kHz_σ_*nonlin*_=0dB). Taking into account the above, we obtain the calibration error σ_*cal*_ equal to 6.19dB (see Multimedia Appendix 5). Finally, knowing the calibration error σ_*cal*_ and assuming the insignificance of the error connected with background noise as well as the gain error, the standard deviations of the frequency nonlinearity error were calculated. The highest value _250Hz_σ_*nonlin*_ was observed at the frequency of 250Hz at the level of 7.28dB (see Multimedia Appendix 5).

No asymmetry of measurement error between the left and right sides was detected in any of the tests, which could raise suspicion concerning incorrect audio balance.

## Discussion

The aim of the research was to identify the measurement error connected with determining the hearing threshold conducted by means of self-administered Web-based pure tone audiometry, as well as identify and analyze factors affecting its value.

The average difference between the value of the hearing threshold determined in series 1 and in series 2 was −1.54dB with standard deviation of 7.88dB and Pearson’s correlation coefficient of .90. Between series 1 and series 3, these values were −1.35dB±10.66dB and .84 respectively. For the average hearing threshold, these values were, for series 2 −1.52dB±5.42dB and .94 respectively, and for series 3, −1.44dB±7.59dB and .89. The results from series 2 are consistent with the results presented by Honeth et al, who determined the correlation coefficient of the average hearing threshold for the group of 72 people at the level of .94 and .93 respectively for the right and left ears [10]. 64 out of 72 people (89%) were examined on the same test set calibrated by the same person.

In series 3, the standard deviation values were most influenced by the error connected with the procedure of determining the hearing threshold, the same as the error in series 2 (σ_*proc*_=6.64dB), the calibration error (σ_*cal*_=6.19dB), and additionally at the frequency of 250Hz, the frequency response nonlinearity error (_250Hz_σ_*nonlin*_=7.28dB).

The error of determining the hearing threshold during examination, which consisted of self-adjustment of the position of the volume slider in 5dB steps, was estimated at the level of σ_*proc*_=6.64dB. The reason for such high error value compared to the error value of the ascending method (σ_*clin*_=4.25dB) may be attributed to the difficulty connected with self-assessment of the hearing threshold. Standard deviations between series 1 and 2 as well as between series 1 and 3 increase together with the increase in the hearing loss (Figure 2). This suggests that the task of self-evaluation of the hearing threshold proves more difficult to perform for subjects with moderate and profound hearing impairment compared to normal hearing subjects. Replacement of this method with the ascending method will contribute to improving the accuracy of the examination. The method of self-evaluation of the hearing threshold was originally chosen as simpler, faster and more flexible. Consequently, it was less monotonous and more attractive for the patient.

The purpose in the calibration was to approximate the 0dBHL level at the examined frequencies. The approximation can be conducted in a number of ways. The reference sound level can be determined independently for each frequency in relation to the hearing threshold of the reference person [10]. In this case, the calibration error at each frequency is burdened with measurement error of the hearing threshold and the difference between the hearing threshold of the reference person and 0dBHL. Another solution, which was adopted in this paper, is to measure the hearing threshold at one frequency, at which differences between the values of the hearing threshold among the population are the smallest and then determine the reference sound level at other frequencies on the basis of the model. Alternatively, calibration can be performed at a number of frequencies and the correctness of the calibration may be assessed based on its accordance with the model. At the same time, in the case of a large discrepancy between the measurement results, the calibration can be repeated by another person or on different headphones. The choice of the optimal method requires further research.

The calibration also involves the problem of selecting a procedure for determining the hearing threshold of the reference person. Calibration is usually conducted by young persons with normal hearing. Therefore, in order to achieve more accurate calibration results, one can use the procedure requiring greater hearing efficiency compared to the procedure used in examination. This study applies calibration consisting in changing the intensity of two sounds with the stable difference of 5dB. This method requires considerable concentration and optimal hearing ability. However, in contrast to the ascending method, it is not burdened with discretization errors.

Calibration error (σ_*cal*_=6.19dB) is connected with the error of hearing threshold assessment σ_*proc_cal*_ and the standard deviation in the hearing threshold of the reference persons σ_*pop*_ at the calibration frequency, ie, 1kHz. The standard deviation of the hearing threshold determined by means of the ascending method among the population of young subjects without prior hearing problems, σ_*pop_asc*_, can be estimated on the basis of literature data [19]. The distribution of the hearing threshold at 1kHz determined for the population of 2490 subjects was approximated with normal distribution. The standard deviation was calculated based on the values of 1st and 3rd quartile at the level of σ_*pop_asc*_=5.6dB. Assuming the standard deviation of the ascending method σ_*clin*_ is at the level of 4.25dB, we obtain σ_*pop*_ at 3.65dB and then σ_*proc_cal*_ at 5.00dB (see Multimedia Appendix 6).

The method of the hearing threshold evaluation used during calibration (σ_*proc_cal*_=5.00dB), despite the possibility of setting the threshold with the accuracy of 1dB step, is characterized by a slightly higher error than in the ascending method σ_*clin*_=4.25dB, whose step equals 5dB. Therefore, accuracy improvement of the Web-based examination can be achieved by modifying the method. An interesting solution is offered by, eg, the Békésy’s method, as it is characterized by lower standard deviation in the test-retest examination compared to the ascending method [17], and additionally it is not burdened with discretization errors.

The frequency nonlinearity error is the difference between the actual values of the reference sound level and the values determined by the model. The greatest value of _250Hz_σ_*nonlin*_=7.28dB was observed at 250Hz with the mean value calculated for all frequencies, excluding the calibration frequency *_mean_*σ_*nonlin*_=4.05dB. The frequency nonlinearity error is connected with different values of Reference Equivalent Threshold Sound Pressure Level (RETSPL) of the headphones used in the examination and the frequency nonlinearity of the sound card. The biggest differences between the RETSPL values occur at low frequencies [20], also in the low frequency range, more often than at mid and high frequencies, frequency distortion of the sound card can be observed, eg, when bass boost option or equalizer settings are enabled. The improvement in accuracy, especially at low frequencies, can be acquired through selection of headphones for which RETSPL values are known. However, in practice this will be hard to achieve for tests carried out at home. An alternative solution would be control determination of the calibration coefficient at low frequencies and verification against the value determined by the model.

Taking into account the significant value of the calibration error and frequency nonlinearity error, it seems interesting to conduct pure-tone audiometry examination on generally available appliances with known electro-acoustic parameters, eg, smartphones. On determining frequency characteristics of the selected smartphone model with bundled headphones, it seems possible to omit the calibration stage and thus obtain more precise examination results.

Web-based pure-tone audiometry can be used without previous training in its conducting. However, it requires the knowledge of basic terms such as the hearing threshold or frequency. On the other hand, an attractive, intuitive and user-friendly interface can largely replace training. Prior to the tests in series 3, patients were instructed on how to perform the test and had performed similar tests before in series 2 under the supervision of an audiologist. The knowledge of the application could lead to reduction in the value of the measurement error.

The obtained results of sensitivity and specificity confirm the possibility of applying Web-based pure-tone audiometry in screening tests. Moreover, Web-based pure-tone audiometry may be used for self-monitoring of hearing, especially if tests are to be conducted under the same conditions. If the same equipment is used, the relative error between subsequent examinations will be reduced by frequency nonlinearity error, and in the case of the same calibration coefficients, relative error will be reduced by the calibration error. Self-monitoring of hearing may become applicable in hearing disorders, such as fluctuating hearing loss, tinnitus, sudden hearing loss, otosclerosis, Ménière’s disease, as well as during treatment with ototoxic drugs. In the future, modifications of the method leading to the decrease in measurement error can broaden the scope of Web-based pure-tone audiometry application.

## Multimedia Appendix 1

Variance of the sum of the two independent random variables.

## Multimedia Appendix 2

The standard deviation for a single measurement of the hearing threshold determined by means of the ascending method.

## Multimedia Appendix 3

The standard deviation for a single measurement of the hearing threshold determined in series 2 and series 3.

## Multimedia Appendix 4

Factors influencing the standard deviation of the measurement error of the hearing threshold in the series 2 and 3.

## Multimedia Appendix 5

The standard deviation of the calibration error and frequency nonlinearity error.

## Multimedia Appendix 6

The standard deviation of the hearing evaluation method used during calibration.

## Multimedia Appendix 7

CONSORT-EHEALTH checklist V1.6.2 [21].

## Abbreviations

dBHL: decibel hearing level
RETSPL: Reference Equivalent Threshold Sound Pressure Level
