# Supplementary figures and images for: Self-Test Web-Based Pure-Tone Audiometry: Validity Evaluation and Measurement Error Analysis
Source: J Med Internet Res. 2013 Apr 12;15(4):e71. doi: 10.2196/jmir.2222 (PMC3636315; doi:10.2196/jmir.2222)

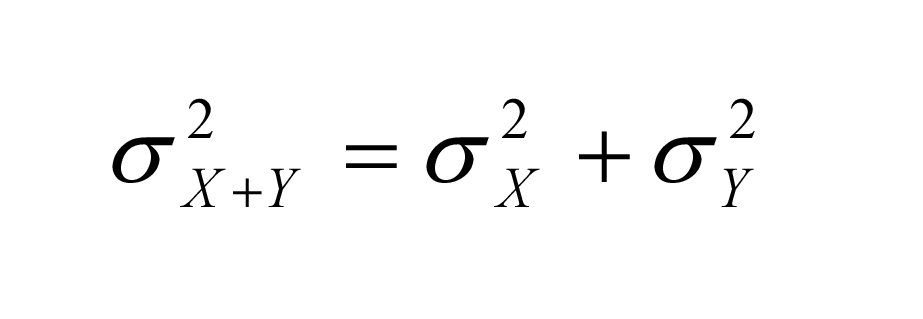

Supplement: Supplementary file 1 [file jmir_v15i4e71_app1.png]

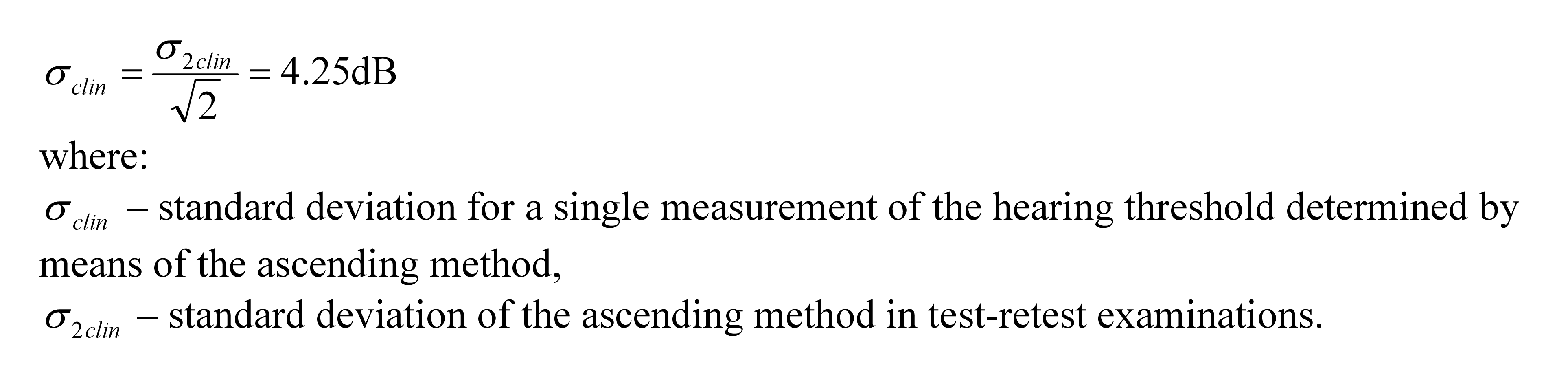

Supplement: Supplementary file 2 [file jmir_v15i4e71_app2.png]

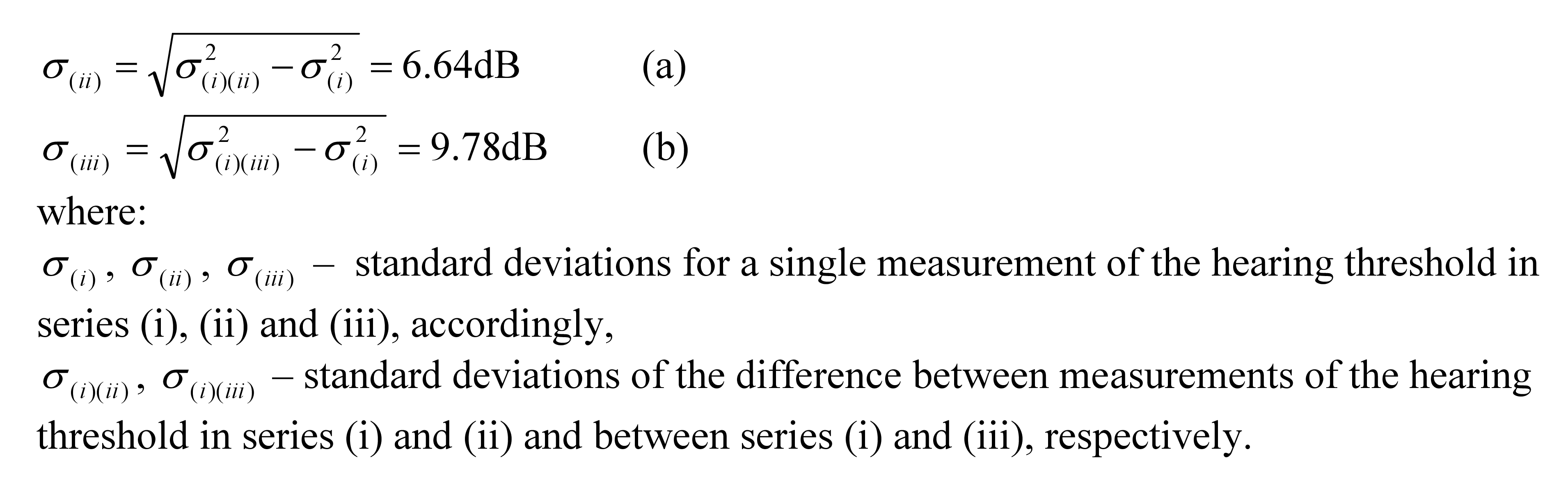

Supplement: Supplementary file 3 [file jmir_v15i4e71_app3.png]

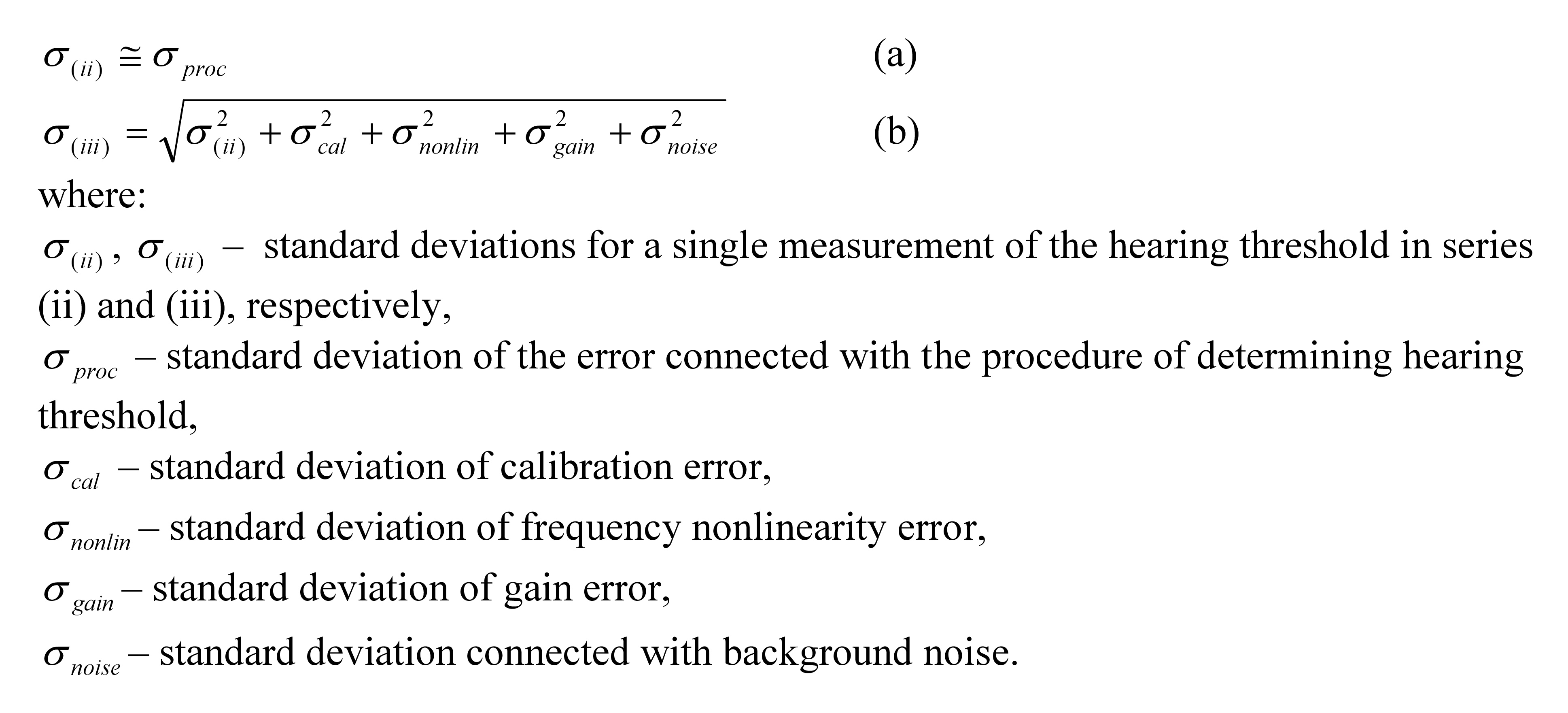

Supplement: Supplementary file 4 [file jmir_v15i4e71_app4.png]

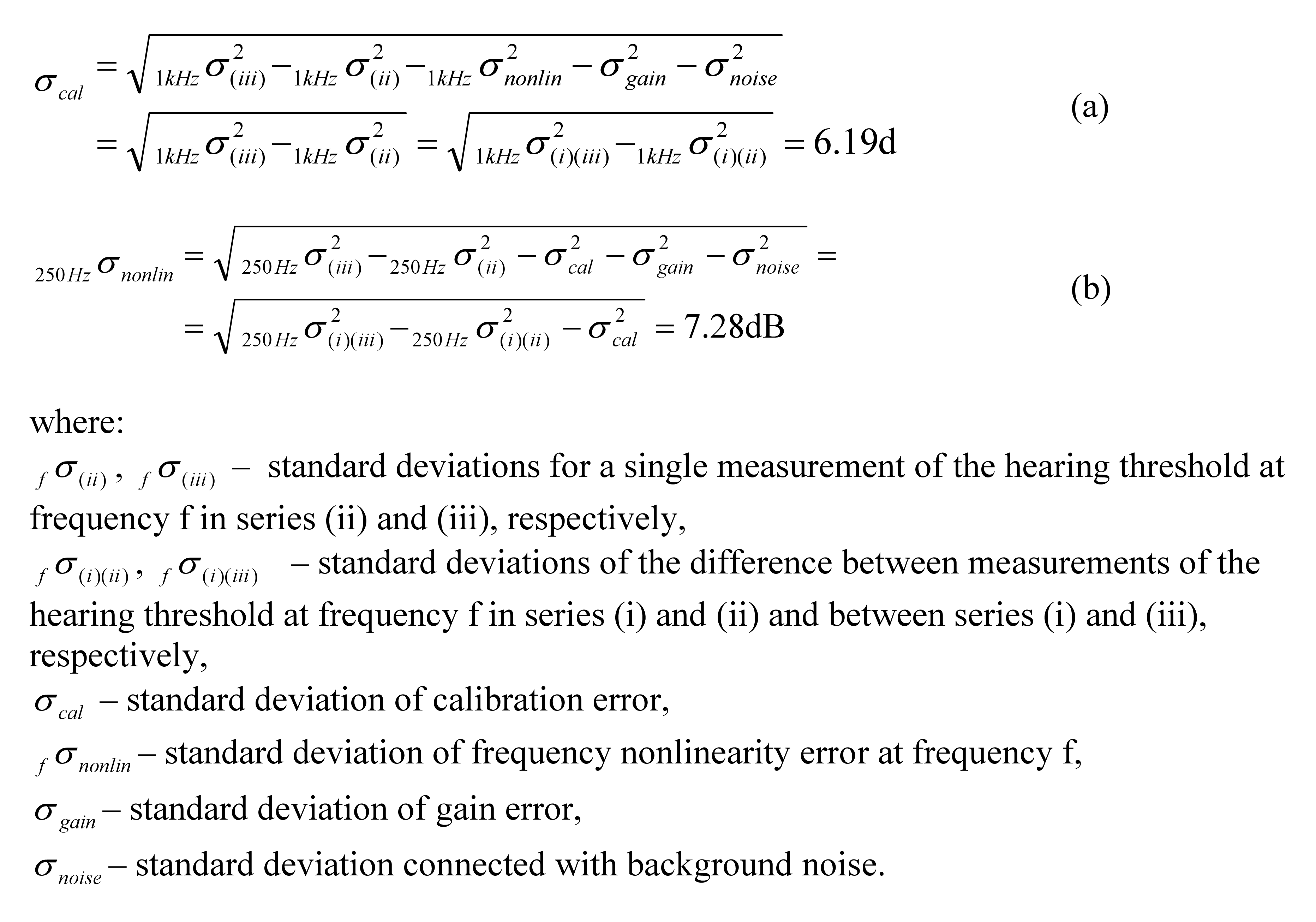

Supplement: Supplementary file 5 [file jmir_v15i4e71_app5.png]

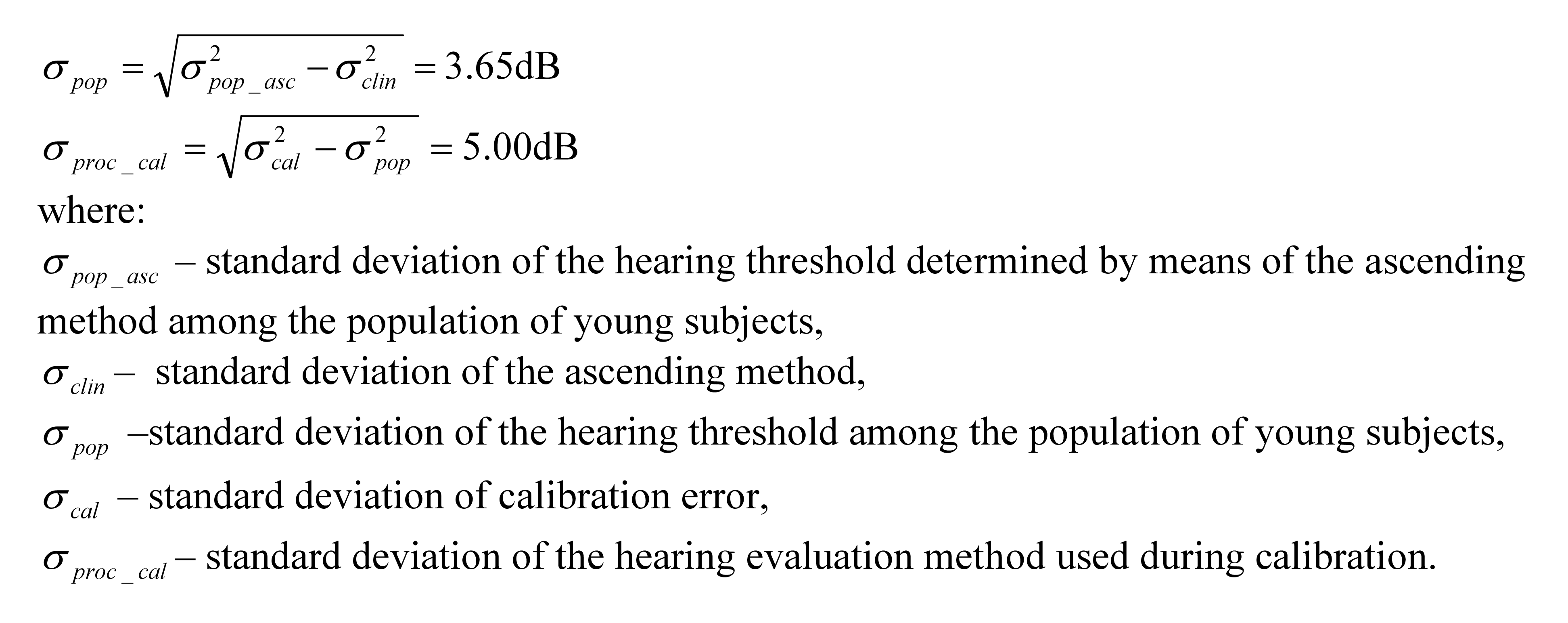

Supplement: Supplementary file 6 [file jmir_v15i4e71_app6.png]
