# Supplementary material for: Self-Test Web-Based Pure-Tone Audiometry: Validity Evaluation and Measurement Error Analysis
Source: J Med Internet Res. 2013 Apr 12;15(4):e71. doi: 10.2196/jmir.2222 (PMC3636315; doi:10.2196/jmir.2222)
Supplement: Supplementary file 7 [file jmir_v15i4e71_app7.pdf]

CONSORT-EHEALTH Checklist V1.6.2 Report

(based on CONSORT-EHEALTH V1.6), available at [http://tinyurl.com/consort-ehealth-v1-6].

2222

Date completed

11/4/2012 10:00:29

by

Marcin Masalski

Self-test web-based pure-tone audiometry: validity evaluation and measurement error analysis

TITLE

1a-i) Identify the mode of delivery in the title

"web-based"

1a-ii) Non-web-based components or important co-interventions in title

None

1a-iii) Primary condition or target group in the title

It would be excessive to mention in the title both the type of examination (pure-tone audiometry) as well as the natural target group for this examination (people with known or suspected hearing problems) which coincides with the research/ trial group (subjects recruited from among audiology outpatient clinic).

ABSTRACT

1b-i) Key features/functionalities/components of the intervention and comparator in the METHODS section of the ABSTRACT

The conducted tests were not Randomized Controlled Trial in nature (see 1a). The paper compares the results of offline tests in series (i) with web-based results from series (ii) and (iii), so it can be assumed that "(i) audiologist-performed tests on a clinical audiometer" is an equivalent of the comparator, while "(ii) self-tests done on a specially calibrated computer under the supervision of an audiologist" and (iii) "self-tests conducted at home" are equivalents of intervention.

1b-ii) Level of human involvement in the METHODS section of the ABSTRACT

Level of human involvement: "(i) audiologist-performed tests..", "(ii) self-tests ... under the supervision of an audiologist", "(iii) self-tests conducted at home".

1b-iii) Open vs. closed, web-based (self-assessment) vs. face-to-face assessments in the METHODS section of the ABSTRACT

The trial was closed, web-based and self-assessed with face-to-face components functioning as a comparator. Subjects were recruited offline: "The research was carried out on the group of 51 subjects selected from among patients of an audiology outpatient clinic".
Face-to-face components: "(i) audiologist-performed tests on a clinical audiometer".

1b-iv) RESULTS section in abstract must contain use data

"From the group of 51 patients examined in the first two series, the series (iii) was self-administered at home by 37 subjects (73%)"

1b-v) CONCLUSIONS/DISCUSSION in abstract for negative trials

The conducted trial was not Randomized Controlled Trial (compare 1a). Its main objective was to determine and analyse the measurement error of the hearing threshold in web-based studies. On the basis of the conducted analysis the following conclusion was formulated:
"The obtained results confirm the possibility of applying web-based pure-tone audiometry in screening tests."

INTRODUCTION

2a-i) Problem and the type of system/solution

The problem: hearing impairment.
Type of solution: stand-alone intervention ("self-administered hearing tests at home", "pure-tone audiometry conducted on a PC ", "self-administered web-based pure tone audiometry").
Patient population and goals of the intervention: General population, in particular / with the focus on persons with hearing problems. However, the target group will depend on measurement error which needs to be evaluated. ("The potential application of pure-tone audiometry conducted on a PC depends on the value of the measurement error. The PC-based test will not substitute the clinical pure-tone audiometry. However, it can be applied for conducting self-administered check-ups, in cases of limited access to clinical devices, e.g. at the general practitioner. Alternatively, it can be used as an initial tele-medical examination combined with a survey, which will help determine the direction of future treatment.")

2a-ii) Scientific background, rationale: What is known about the (type of) system

"The remote examinations of hearing conducted with the use of PC have been carried out for about 10 years [1]. These examinations can be divided into surveys [2, 3, 4] and examinations done with the use of sound signals. The sound signals can be generated through a dedicated external device connected to a PC, usually an audiometer [5, 6] or a PC sound card [3, 4, 7, 8, 9, 10, 11, 12]. Among hearing examinations we can distinguish standard screening tests [3, 7, 11, 12] and examinations such as pure-tone audiometry [4, 5, 6, 8, 9, 10], which provide additional information.
The hearing screening tests done remotely with the use of a PC sound card are most often in the form on a speech-in-noise test [3, 7, 11, 12]. The speech-in-noise test is preferred over survey [3] as it improves the detection of hearing impairment among the population [7], especially after introducing a low-pass noise [12]. The evaluation of validity of pure-tone audiometry conducted in similar conditions, i.e. with the use of a PC sound card and ordinary headphones [8, 9, 10] is ambiguous and depends on the adopted solutions, which include e.g. calibration, hearing threshold evaluation method, presence or lack of a person supervising the test. In the supervised tests the mean error concerning the determination of a hearing threshold on a specially constructed and calibrated PC-based device was below 2.3dB [8], in unsupervised tests conducted after computer calibration performed by a person with a good hearing the greatest error occurred at the frequency of 2 and 4kHz and was −5.6dB and −5.1dB respectively, with standard deviation of 8.29dB and 6.9dB [10]. In unsupervised tests conducted without calibration the maximum mean difference occurred at the frequency of 500Hz and was 11.3dB [9]."

METHODS

3a) CONSORT: Description of trial design (such as parallel, factorial) including allocation ratio

"The aim of the research was to identify the measurement error connected with determining the hearing threshold conducted by means of self-administered web-based pure tone audiometry, as well as identify and analyse factors affecting its value."

3b) CONSORT: Important changes to methods after trial commencement (such as eligibility criteria), with reasons

None

3b-i) Bug fixes, Downtimes, Content Changes

None

4a) CONSORT: Eligibility criteria for participants

"The research participants were selected from among the patients of an audiology outpatient clinic. The qualification criterion was the willingness to participate in the research, owning a PC, basic skills to operate it and having an email account."

4a-i) Computer / Internet literacy

"The qualification criterion was ... owning a PC, basic skills to operate it and having an email account."

4a-ii) Open vs. closed, web-based vs. face-to-face assessments:
Closed, web-based with face-to-face components:
"The hearing threshold evaluation was made in three series: (i) audiologist-performed tests on clinical audiometer at an audiology outpatient clinic (ii) self-administered web-based tests on a specially calibrated computer at an audiology outpatient clinic under the supervision of an audiologist and (iii) self-administered web-based tests conducted at home... The research participants were selected from among the patients of an audiology outpatient clinic. The qualification criterion was the willingness to participate in the research, owning a PC, basic skills to operate it and having an email account."
4a-iii) Information giving during recruitment
The patients of the audiology outpatient clinic received a leaflet with information on the trial. The leaflet, together with its translation, is available in the multimedia appendices.
Pacjenci poradni audiologicznej otrzymywali ulotkę z informacjami dotyczącymi eksperymentu (ang. trial). Ulotka i jej tłumaczenie została załączona jako dodatek multimedialny.
4b) CONSORT: Settings and locations where the data were collected
serie(i): "All tests from series (i) were made in an acoustic cabin with the use of clinical audiometer..."
serie(ii): "All the examinations in series (ii) were conducted on a notebook Dell Vostro 1320 with operational system Microsoft Windows 7 and headphones Technics RP-F290 placed in an acoustic cabin of an audiology outpatient clinic and connected to the Internet. "
serie(iii): "Examinations in series (iii) were conducted by the patients themselves on their own personal computers at home."
4b-i) Report if outcomes were (self-)assessed through online questionnaires
The outcomes were not assessed through online questionnaires.
4b-ii) Report how institutional affiliations are displayed
Affiliations of universities which conducted the project were placed in the top left corner of the website.
5) CONSORT: Describe the interventions for each group with sufficient details to allow replication, including how and when they were actually administered
5-i) Mention names, credential, affiliations of the developers, sponsors, and owners
"The author of this article is the owner of the Internet portal e-audiologia.pl, on the basis of which the tests above were conducted."
“The trial was financed by Wrocław Medical University as part of Research Grant for Young Scientists. Grant No. 38/Pbmn.”
5-ii) Describe the history/development process
Web-based hearing tests were conducted on the basis of applets written in the Java language and run via Internet browser. Next, test results are transferred using applet/servlet technology and stored in MySQL database. The first author of this paper is the designer and manufacturer of this website in whole, excluding the graphics.
First versions of the software were created in 2006 and have been regularly improved since then according to the observations of the first author of this paper as well as opinions of other doctors and patients.
5-iii) Revisions and updating
No major changes were introduced during the trial.
5-iv) Quality assurance methods
Software testing.
5-v) Ensure replicability by publishing the source code, and/or providing screenshots/screen-capture video, and/or providing flowcharts of the algorithms used
The instruction on how to conduct test in series (ii) and (iii) features screenshots together with translations of their most important elements available in multimedia appendices.
5-vi) Digital preservation
URL of the application:
http://www.e-audiologia.pl/JMIRms2222/index.jsp?stan=strona_glowna
http://www.webcitation.org/6Bv10Jbqp
The demo version of the web-based pure tone audiometry:
http://www.e-audiologia.pl/JMIRms2222/index.jsp?stan=audiometria_tonalna_demo
http://www.webcitation.org/6Bv1LBvwF
As webcitation does not archive applets properly, applet screenshots are available in "The instruction for web-based tests" in multimedia appendices.
5-vii) Access
Before starting the tests in series (ii) patients has to create their own accounts on www.e-audiologia.pl website by typing in their login and password, and then activating their account using the activation link which was sent via e-mail address given at registration. Next steps of the procedure were as described in the instruction available in multimedia appendices. Additionally, the data for a demo account are provided (5-vi), which allows to preview the test without registration.
5-viii) Mode of delivery, features/functionalities/components of the intervention and comparator, and the theoretical framework
Mode of delivery, features/functionalities/components of the comparator and the theoretical framework (series i):
- Starting with: "All tests from series (i) were made in an acoustic cabin with the use of clinical audiometer Interacoustic AD229e and headphones TDH-39..."
- instructional strategy: audiologist-guided task,
- mode of delivery, delivery platform: offline

Mode of delivery, features/functionalities/components of the intervention and the theoretical framework (series ii and iii):
- Starting with: "The examinations in series (ii) and (iii) were conducted on personal computers following their calibration",
- instructional strategy: self-assessment task,
- mode of delivery: web page,
- delivery platform: PC, speed of Internet access - unknown,
- the whole application was developed by the first author,
- the application allows users to track results of previous examinations,
- the application allows users to obtain information via e-mail or phone, but this option was not used in the trial aplikacja umożliwia użytkownikom uzyskanie informacji zwrotnej przez email lub telefonicznie, ale opcje ta nie była wykorzystywana w badaniu (ang. trial),
- page design principles, average amount of text on pages, presence of hyperlinks to other resources: see multimedia appendices, "Instruction for web-based tests" (multimedia appendices).

5-ix) Describe use parameters
"The trial participants were instructed to conduct the test at home in a quiet place, preferably in the evening or at night. Moreover, they were informed that the calibration should be performed by a person up to 35 years old, with no previous hearing problems.”
Also compare "Instruction for the web-based test" available in multimedia appendices.
5-x) Clarify the level of human involvement

In the series (i): "The hearing threshold in pure-tone audiometry was determined with the use of the ascending method, according to ISO 8253-1:2010".
In the series (ii): "Examinations were supervised by an audiologist, whose task was to train a patient and detect his\her mistakes, e.g. changing the sides of headphones, omission of the frequency or accidental marking of the threshold which was significantly different from the actual one."
In the series (iii): "Examinations in series (iii) were conducted by the patients themselves on their own personal computers at home."

5-xi) Report any prompts/reminders used

"The examinations in series (i) and (ii) were conducted on the same day, and examinations from series (iii) were conducted up to 196 days later (median of 9 days)."
"In the case of failure to conduct tests in series (iii), the patients were again requested by phone to perform the test after about 2-3 weeks and then reminded by e-mail after about 1 month."

5-xii) Describe any co-interventions (incl. training/support)

"Examinations were supervised by an audiologist, whose task was to train a patient and detect his\her mistakes, e.g. changing the sides of headphones, omission of the frequency or accidental marking of the threshold which was significantly different from the actual one."

6a) CONSORT: Completely defined pre-specified primary and secondary outcome measures, including how and when they were assessed

Despite the fact that the research is not a Randomized Controlled Trial, primary outcome measures can be assumed to be the values of the hearing thresholds determined in series (i), (ii) and (iii). No secondary outcome measures were conducted.

6a-i) Online questionnaires: describe if they were validated for online use and apply CHERRIES items to describe how the questionnaires were designed/deployed

No online questionnaires were used.

6a-ii) Describe whether and how “use” (including intensity of use/dosage) was defined/measured/monitored

In order to carry out tests in a single series the patient needed to log in once only. Patients could stop the test and continue after logging in again. Patients’ logging activity was not registered.

6a-iii) Describe whether, how, and when qualitative feedback from participants was obtained

No feedback was collected from the patients.

6b) CONSORT: Any changes to trial outcomes after the trial commenced, with reasons

No changes were introduced during the trial.

7a) CONSORT: How sample size was determined

7a-i) Describe whether and how expected attrition was taken into account when calculating the sample size

The aim of the research was to identify the measurement error connected with determining the hearing threshold conducted by means of self-administered web-based pure tone audiometry. It has been assumed that the confidence interval of the determined values of standard deviations should equal about -+1dB at statistical significance level of P=.05. If we assume the standard deviation of the population at the level of 4.25dB resulting from the value of the deviation in test-retest examination conducted using the ascending method (literature data) we receive the required number of measurements at the level of 35. In series (i) and (ii) participated 51 subjects, and in series (iii) 37 subjects conducted the test.

7b) CONSORT: When applicable, explanation of any interim analyses and stopping guidelines

No interim analyses were performed. Stopping guidelines - compare 7a-i)

8a) CONSORT: Method used to generate the random allocation sequence

Not applicable - no randomization.

8b) CONSORT: Type of randomisation; details of any restriction (such as blocking and block size)

Not applicable - no randomization.

9) CONSORT: Mechanism used to implement the random allocation sequence (such as sequentially numbered containers), describing any steps taken to conceal the sequence until interventions were assigned

Not applicable - no randomization.

10) CONSORT: Who generated the random allocation sequence, who enrolled participants, and who assigned participants to interventions

Not applicable - no randomization.

11a) CONSORT: Blinding - If done, who was blinded after assignment to interventions (for example, participants, care providers, those assessing outcomes) and how

11a-i) Specify who was blinded, and who wasn’t

The participants could compare the results obtained in each series, the technicians were not aware of the results in series (iii). The whole statistical analysis was conducted by the same person (the first author of the paper).

11a-ii) Discuss e.g., whether participants knew which intervention was the “intervention of interest” and which one was the “comparator”

The conducted study was not Randomized Controlled Trial (see 1a). The participants were of course aware that the test results from series (i) would be compared with those from series (ii) and (iii).

11b) CONSORT: If relevant, description of the similarity of interventions

The research in all three series consisted of determining the subjective hearing threshold by means of pure-tone audiometry.

12a) CONSORT: Statistical methods used to compare groups for primary and secondary outcomes

"The hearing threshold determined in series (i) was compared to hearing thresholds from series (ii) and (iii). Figure 1 presents relationships between the series: the relationship between hearing thresholds with division into frequencies, the relationship between total hearing thresholds and that between the mean hearing thresholds calculated on the basis of the value obtained at all the examined frequencies. The mean difference between thresholds in series, its standard deviation, Pearson’s correlation coefficient and linear estimators (table 2) were calculated for the relationships described above. Linear estimators were determined for Deming's regression because the explanatory variable, which constitutes the hearing threshold in series (i), is also burdened with measurement error. "

12a-i) Imputation techniques to deal with attrition / missing values

When comparing series (i) and (ii) the results from all the test participants were taken into account (51 subjects). By the same token, when comparing series (i) and (iii) all the subjects who took part in both series were taken into account (37 subjects).
“For hearing threshold below 0dBHL-RP , the value was assumed to be 0dBHL-RP
“If there was no response at a given frequency, measurement was not taken into account for further calculations.”

12b) CONSORT: Methods for additional analyses, such as subgroup analyses and adjusted analyses

An analysis of the measurement error was conducted in series (ii) and (iii) based on the mathematical properties of variances, and in particular the variance of the sum of two independent variables.

RESULTS

13a) CONSORT: For each group, the numbers of participants who were randomly assigned, received intended treatment, and were analysed for the primary outcome

No randomization was performed. The number of subjects in each series was given in the chapter "Methods":"In series (i) and (ii) 51 participants, aged between 11 – 60 (the median age of 34) underwent examination...From the group of 51 patients examined in the first two series, the series (iii) was self-administered at home by 37 subjects (73%)."

13b) CONSORT: For each group, losses and exclusions after randomisation, together with reasons

All (51) patients qualified to take part in the trial were tested in series (i) and (ii).

13b-i) Attrition diagram

After about 5 months from the end of the trial, an additional web-based hearing tests were noticed on the accounts of 13 out of 51 test participants (25.5%).

14a) CONSORT: Dates defining the periods of recruitment and follow-up

recruitment and examination in series (i) and (ii): 02.08.2011 - 04.04.2012
examination in series (iii): 04.08.2011 - 13.05.2012

14a-i) Indicate if critical "secular events" fell into the study period

No critical events were observed during the trial.

14b) CONSORT: Why the trial ended or was stopped (early)

The trial was finished as the sufficient number of patients took part in its all three series.

15) CONSORT: A table showing baseline demographic and clinical characteristics for each group

"In series (i) and (ii) 51 participants (32 men, 19 women), aged between 11 – 60 (the median age of 34) underwent examination...102 ears were examined from which 45 (44%) were ears without a hearing loss i.e. with the hearing threshold of 25dBHL and less, 17 (16.7%) were ears with hearing impairment below 40dBHL, 31 (30.4%) with hearing impairment above 40dBHL and below 70dBHL and 9 (8.8%) above 70dBHL."

15-i) Report demographics associated with digital divide issues

"In series (i) and (ii) 51 participants (32 men, 19 women), aged between 11 – 60 (the median age of 34) underwent examination...The qualification criterion was the willingness to participate in the research, owning a PC, basic skills to operate it and having an email account." Education and social-economic status of the subjects were not taken into account.

16a) CONSORT: For each group, number of participants (denominator) included in each analysis and whether the analysis was by original assigned groups

16-i) Report multiple "denominators" and provide definitions

"From the group of 51 patients examined in the first two series, the series (iii) was self-administered at home by 37 subjects (73%). The examinations in series (i) and (ii) were conducted on the same day, and examinations from series (iii) were conducted up to 196 days later (median of 9 days)."

16-ii) Primary analysis should be intent-to-treat

All trial participants who started tests in each series finished them. No single participant performed tests partially.

17a) CONSORT: For each primary and secondary outcome, results for each group, and the estimated effect size and its precision (such as 95% confidence interval)

The study is not Randomized Clinical Trial, and its objective is the analysis of the measurement error. The value of effect size and its precision are not applicable.

"The mean difference in the hearing threshold between series (i) and (ii) and between series (i) and (iii) was −1.54dB and −1.34dB respectively, with standard deviation 7.88dB and 10.66dB respectively, and Pearson's correlation coefficients 0.90 and 0.84 respectively. In both comparisons the lowest values of Pearson's correlation coefficient were obtained for the frequency of 250Hz at the level of 0.88 and 0.69 respectively. The highest value of the standard deviation occurred at the frequency of 8kHz (8.88dB) and 500Hz (12.03dB) respectively. The Pearson's correlation coefficients calculated for the mean threshold were at the level of 0.94 and 0.89 respectively (table 1).

The mean difference between thresholds in series, its standard deviation, Pearson's correlation coefficient and linear estimators of Deming's regression were determined for the hearing loss below 40dBHL as well as greater than or equal to 40dBHL (table 2)..."

The confidence intervals values are given in Table 1.

17a-i) Presentation of process outcomes such as metrics of use and intensity of use

The trial was conducted once only. The time spent on calibration and the test was not limited. The time of calibration and the test was not recorded.

"The reference person could listen to the sound for unlimited time, adjusting the volume using the slider with 1dB step any number of times, in order to finally confirm the selected level with a button...Patients could listen to the sound for unlimited time and change the intensity of the presented sound signal by themselves using a slider with 5dB step any number of times, and then confirmed the chosen level with a button."

17b) CONSORT: For binary outcomes, presentation of both absolute and relative effect sizes is recommended

There was no binary outcome in the trial.

18) CONSORT: Results of any other analyses performed, including subgroup analyses and adjusted analyses, distinguishing pre-specified from exploratory

Results of the measurement error analysis:

"In series (iii) the standard deviation values were most influenced by: the error connected with the procedure of determining the hearing threshold, the same as the error in series (ii) (6.64dB), the calibration error (6.19dB) and additionally at the frequency of 250Hz, the frequency response nonlinearity error (7.28dB)."

18-i) Subgroup analysis of comparing only users

All the trial participants were users.

19) CONSORT: All important harms or unintended effects in each group

No important harm or other side effects were detected.

19-i) Include privacy breaches, technical problems

After registering an account, patients were sent an activation link via e-mail. In order to reduce possible delays which were sometimes connected with sending the link via e-mail, a technician could speed up the procedure of activating the account by typing in a specially prepared address in the browser window, which contained coded information about activation.

19-ii) Include qualitative feedback from participants or observations from staff/researchers

Observations of people supervising the project were not recorded. However, greater difficulties were observed in determining the hearing threshold of elderly people with more serious hearing loss and it took them longer to conduct the test. This is reflected in the fact that the measurement error increased together with the increase in the hearing loss.

DISCUSSION

20) CONSORT: Trial limitations, addressing sources of potential bias, imprecision, multiplicity of analyses

20-i) Typical limitations in ehealth trials

"Prior to the tests in series (iii) patients were instructed on how to perform the test and had performed similar tests before in series (ii) under the supervision of an audiologist. The knowledge of the application could lead to reduction in the value of the measurement error."

21) CONSORT: Generalisability (external validity, applicability) of the trial findings

21-i) Generalizability to other populations

"Web-based pure-tone audiometry can be used without previous training in its conducting. However, it requires the knowledge of basic terms such as the hearing threshold or frequency. On the other hand, an attractive, intuitive and user-friendly interface can largely replace training."

21-ii) Discuss if there were elements in the RCT that would be different in a routine application setting

Trial participants were instructed by the audiologist (technician) on how to perform the test, while other Internet users have no direct contact with the instructor.

22) CONSORT: Interpretation consistent with results, balancing benefits and harms, and considering other relevant evidence

22-i) Restate study questions and summarize the answers suggested by the data, starting with primary outcomes and process outcomes (use)

Study questions: "The aim of the research was to identify the measurement error connected with determining the hearing threshold conducted by means of self-administered web-based pure tone audiometry, as well as identify and analyse factors affecting its value."

Answers suggested by the data: "In series (iii) the standard deviation values were most influenced by: the error connected with the procedure of determining the hearing threshold, the same as the error in series (ii) (6.64dB), the calibration error (6.19dB) and additionally at the frequency of 250Hz, the frequency response nonlinearity error (7.28dB)."

"The error of determining the hearing threshold during examination, which consisted in self-adjustment of the position of the volume slider in 5dB steps, was estimated at the level of 6.64dB. The reason for such high error value compared to the error value of the ascending method (4.25dB) may be attributed to the difficulty connected with self-assessment of the hearing threshold..."

"Calibration error (6.19dB) is connected with the error of hearing threshold assessment and the standard deviation in the hearing threshold of the reference persons at the calibration frequency i.e. 1 kHz. The method of the hearing threshold evaluation used during calibration (5.55), despite the possibility of setting the threshold with the accuracy of 1dB step, is characterised by a slightly higher error than in the ascending method (4.25), whose step equals 5dB. Therefore, accuracy improvement of the web-based examination can be achieved by modifying the method."

"The frequency nonlinearity error is the difference between the actual values of the reference sound level and the values determined by the model. The greatest value of 7.28dB was observed at 250Hz with the mean value calculated for all frequencies, excluding the calibration frequency 4.05dB..... The improvement in accuracy, especially at low frequencies, can be acquired through selection of headphones for which RETSPL values are known. However, in practice this will be hard to achieve for tests carried out at home. An alternative solution would be control determination of the calibration coefficient at low frequencies and verification against the value determined by the model."

22-ii) Highlight unanswered new questions, suggest future research

"The purpose in the calibration was to approximate the 0dBHL level at the examined frequencies. The approximation can be conducted in a number of ways... The choice of the optimal method requires further research."

"The method of the hearing threshold evaluation used during calibration (5.55dB), despite the possibility of setting the threshold with the accuracy of 1dB step, is characterised by a slightly higher error than in the ascending method 4.25dB, whose step equals 5dB. Therefore, accuracy improvement of the web-based examination can be achieved by modifying the method. An interesting solution is offered by e.g. the Bekesy's method, as it is characterised by lower standard deviation in the test-retest examination compared to the ascending method [17] and additionally it is not burdened with discretization error."

"The improvement in accuracy, especially at low frequencies, can be acquired through selection of headphones for which RETSPL values are known. However, in practice this will be hard to achieve for tests carried out at home. An alternative solution would be control determination of the calibration coefficient at low frequencies and verification against the value determined by the model."

Other information

23) CONSORT: Registration number and name of trial registry

Trial was not registered. Refer to 1a).

24) CONSORT: Where the full trial protocol can be accessed, if available

Full trial protocol is not available. Refer to 1a).

25) CONSORT: Sources of funding and other support (such as supply of drugs), role of funders

"The trial was financed by the Wrocław Medical University as part of Research Grant for Young Scientists. Grant No. 38/Pbmn."

X26-i) Comment on ethics committee approval

Consent of Bioethical Committee to conduct the trial has been obtained.

x26-ii) Outline informed consent procedures

Consent for participation in the trial was collected offline. The translation of the consent form is available in the additional material.

X26-iii) Safety and security procedures

In order to protect the patients from loud noises they were instructed to close all other sound-generating applications before starting the test. The hearing test was initiated at 40dB above the reference sound level.

X27-i) State the relation of the study team towards the system being evaluated

"The author of this article is the owner of the Internet portal e-audiologia.pl, on the basis of which the tests above were conducted."
